# Lower limb joint replacement in rheumatoid arthritis

**DOI:** 10.1186/1749-799X-7-27

**Published:** 2012-06-14

**Authors:** Nicholas D Clement, Stephen J Breusch, Leela C Biant

**Affiliations:** 1 Orthopaedic Research Fellow, Royal Infirmary of Edinburgh, Little France, EH16 4SA, UK; 2 Orthopaedic Surgery, University of Heidelberg, Heidelberg, Germany; 3 Orthopaedic Surgeon, Royal Infirmary of Edinburgh, Little France, EH16 4SA, UK

## Abstract

**Introduction:**

There is limited literature regarding the peri-operative and surgical management of patients with rheumatoid disease undergoing lower limb arthroplasty. This review article summarises factors involved in the peri-operative management of major lower limb arthroplasty surgery for patients with rheumatoid arthritis.

**Methods:**

We performed a search of the medical literature, using the PubMed search engine (http://www.pubmed.gov). We used the following terms: ‘rheumatoid’ ‘replacement’ ‘arthroplasty’ and ‘outcome’.

**Findings:**

The patient should be optimised pre-operatively using a multidisciplinary approach. The continued use of methotrexate does not increase infection risk, and aids recovery. Biologic agents should be stopped pre-operatively due the increased infection rate. Patients should be made aware of the increased risk of infection and periprosthetic fracture rates associated with their disease. The surgical sequence is commonly hip, knee and then ankle. Cemented total hip replacement (THR) and total knee replacement (TKR) have superior survival rates over uncemented components. The evidence is not clear regarding a cruciate sacrificing versus retaining in TKR, but a cruciate sacrificing component limits the risk early instability and potential revision. Patella resurfacing as part of a TKR is associated with improved outcomes. The results of total ankle replacement remain inferior to THR and TKR. RA patients achieve equivalent pain relief, but their rehabilitation is slower and their functional outcome is not as good. However, the key to managing these complicated patients is to work as part of a multidisciplinary team to optimise their outcome.

## Introduction

Rheumatoid arthritis (RA) is a chronic systemic connective tissue disease and is the third most common indication for lower limb joint replacement in Northern Europe and North America [1]. The aetiology of the disease remains unclear but there are strong associations with Human Leukocyte Antigens (DRB1) [2]. The prognosis is poor with 80% of patients being disabled 20 years from primary diagnosis [3]. The medical treatment of RA has improved during the last 25 years, which is reflected by a 40% decrease in the rate of hip and knee surgery since a peak that was observed in the mid 1990s [4]. Anaemia, raised erythrocyte sedimentation rate and a high disease activity score have all been identified as risk factors for requirement of large joint arthroplasty [5]. Seventeen percent of patients with RA undergo an orthopaedic intervention within 5 years of initial diagnosis [5]. Over a third of patients will need a major joint replacement, of which the majority will receive a total hip or knee replacement (THR and TKR) [4]. This review article summarises factors involved in the peri-operative management of major lower limb arthroplasty surgery for patients with RA.

## Methods of literature search

We searched the PubMed.gov© [6] electronic database for studies published in English between 1960 and 2011. Our defined search term was: ‘rheumatoid’ ‘replacement’ ‘arthroplasty’ and ‘outcome’. This identified 669 eligible articles. All 669 abstracts were reviewed and those matching the inclusion criteria were included, and the full paper was retrieved.

The inclusion criteria were:

1. Articles reporting pre-operative management of patients with RA receiving an orthopaedic intervention

2. Articles reporting the survivorship and/or functional outcome and/or complications of primary total hip/knee/ankle replacements in patients with RA

3. Articles reporting the survivorship and/or functional outcome and/or complications of revision total hip/knee/ankle replacements in patients with RA

4. Articles reporting the rehabilitation of patients with RA after total hip/knee/ankle replacements

Due to the insufficiency of published literature regarding arthroplasty in the patients with RA further literature searches were executed. This was only performed when there was insufficient data to draw a conclusion upon the question being addressed e.g. use of tumour necrosis factor alpha (TNFα) drugs in patients with RA undergoing arthroplasty surgery.

## Pre-operative assessment

Pre-operative: history, examination and investigations need to be comprehensive (Table 1) [7].

**Table 1:** Systemic preoperative assessment of the rheumatoid patient

**History**	**Examination**	**Investigations**
Disease onset	Complete medical	Full blood count
Pattern and sequence	Joint inflammation	Urea & creatinine
Presences and persistent joint swelling	Joint damage and range of motion	Electrolytes
Pain (site, severity, duration)	Soft tissue integrity	Liver function tests
Morning stiffness	Extra-articular features	Chest radiograph
Functional limitations	Grip strength	Cervical spine radiograph
Non-articular features	General health	Electrocardiogram
Psychological features	Dental inspection	Urine dipstick +/− culture
Systemic features	Neurological assessment	Pulmonary function tests
Review of all systems		Echocardiogram (limiting cardiac pathology)
Prior anaesthetic and surgery		
Drugs and allergies		Airway assessment

Eighty percent of RA patients have cervical spine involvement. Thirty percent have instability of the cervical spine, half of whom are asymptomatic [8,9]. Subluxation of the atlanto-axial joint, due to the destruction of the transverse ligament by inflammatory pannus, is defined as a distance of >3 mm between the anterior aspect of the atlas and dens on a plain lateral cervical spine radiograph [7]. Clinical symptoms of occipital headache, weakness of limbs, bladder and bowel dysfunction, and long track signs should alert the clinician to such pathology. Computerised tomography (CT) may be helpful to assess the extent of subluxation [10].

## Immunosuppressants

Steroids are used as a therapeutic bridge to control the symptoms until the disease modifying anti rheumatic drugs (DMARDs) take effect. If a patient has used long-term steroids, an increased dose should be given in times of stress to prevent an Addisionian crisis. Use of steroids in the peri-operative period for general surgical procedures increases the infection rate and impedes wound healing [11]. There is, however, no published literature regarding the risk of steroid use in the peri-operative period for arthroplasty surgery.

Methotrexate is a commonly used DMARD and has been shown to improve symptoms and slow radiographic progression of joint destruction [12]. There is a single prospective randomised control trial, which recruited 388 patients undergoing elective surgery who were randomised to either cease or continue with methotrexate [13]. They demonstrated a 2% infection rate in those who continued methotrexate, with a decreased complication rate and number of flares of their rheumatoid disease. Those who stopped the methotrexate, had a 15% infection rate. Hence it would seem safe and beneficial for the patient to continue their methotrexate peri-operatively, and may aid their post-operative recovery.

Newer targeted immunotherapy such as TNFα antagonists are more effective in disease control with slowing of radiographic joint destruction [14]. The evidence as to whether these drugs should be continued or stopped during orthopaedic procedures is limited. One study of 31 patients undergoing foot and ankle surgery demonstrated no difference in the infection rate if patients continued with their TNFα prescription [15]. A larger retrospective study of 128 patients undergoing major orthopaedic surgery revealed an increased infection risk in those who remained on TNFα antagonists (odds ratio 21.8), and an associated increased risk of deep vein thrombosis (odds ratio 2.8) [16].

## Surgical sequence

Wilkinson et al suggested addressing lower limb arthropathy before the upper limb; with the hypothesis that prior fragile upper limb interventions may be damaged by mobilisation on crutches after lower limb surgery [7]. The surgical sequence they recommended was: forefoot, hip, knee, hind foot and then ankle, which they deemed the order of “reliability” of the procedures. Constructing a base on which you can build would be logical, the “reliability” of different procedures is arguable and individual patient assessment may dictate a different protocol. Hindfoot fusion may necessitate plaster immobilization, and could be considered at an earlier stage. Restoration of the correct femoral alignment and length with a THR precedes the TKR to allow correct implant alignment and rotation. Significant joint stiffness and/or contracture at adjacent or bilateral joints may be optimally addressed by simultaneous arthroplasty surgery. Pre-operative long leg standing alignment radiographs and a CT scan for assessment of soft tissue integrity and bone loss can help plan surgery.

## Total hip replacement

Technical challenges of performing THR in patients with RA are mainly due to bone loss, osteopenia and protrusio acetabuli. These patients are not suitable for hip resurfacing because of the risk of secondary osteoporosis [17].

Until recently there has been little evidence to support the use of cemented over uncemented THR. Chmell et al reviewed 39 patients with juvenile rheumatoid disease (66 hips) who received a cemented THR with a mean follow of 15.1 years [18]. They report a stem survival of 85% and a cup survival of 70%, for various implant designs. Creighton et al reviewed 75 patients (106 hips) all of whom received a cemented prosthesis and revealed a stem survival of 98% and cup survival of 92% at 10 years [19]. They also demonstrated an association of cup loosening with younger patients. Jana et al, using an uncemented stem in 64 patients (82 hips) for juvenile RA, reported a survival of 98.1% at 11 years. However, various cemented and uncemented cups were used. Analysis of 2,557 primary THR using various implants for patients with RA from the Finnish arthroplasty register found the best survival to be with uncemented proximally circumferentially porous-coated stems (89% survival at 15 years) and cemented all-polyethylene cups (80% survival at 15 years) [20]. However, more recent data from the Norwegian arthroplasty register suggested that cemented THR was superior to uncemented THR, with a 10 year survival of 89% and 81% respectively [21].

**Figure 1:**
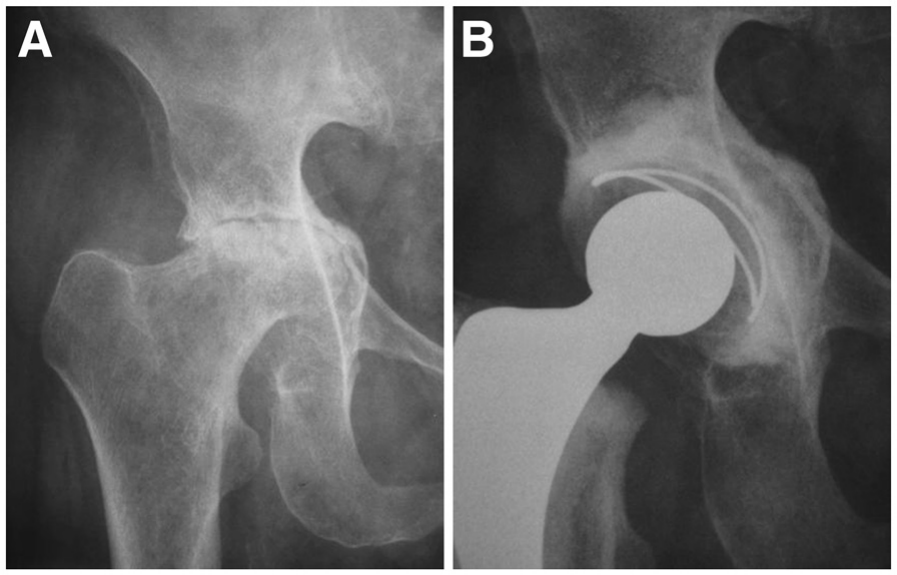
Grade II protrusio acetabuli (A) in a female that underwent THR with medial bone graft and restoration of the center of rotation (B).

**Figure 2:**
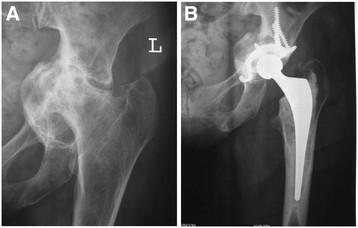
Grade III protrusio acetabuli (A) with cage augmentation and medial bone graft (B).

Protrusio acetabuli is a common occurrence in the rheumatoid hip and technical difficulties can be encountered due to medial wall deficiency. Two grading systems are used; that of Charnley [22], relative to the ilio-pectineal line, and more commonly Hirst et al. [23], relative to the ilio-ischial line (Table 2). Hirst also described the Wrightington technique for bone grafting the acetabular floor, using 2 mm discs cut from the dislocated femoral head, which are molded using a dome pusher to conform to the acetabular floor. Restoration of the center of rotation lateral to Köhler’s teardrop is essential (Figure 1). To further improve cement fixation 6 mm holes may be drilled around the periphery of the acetabulum. The cement is placed directly onto the floor graft with insertion of the cup. More extensive acetabular destruction in Grade III protrusio may require a cage and additional bone grafting to prevent early failure (Figure 2).

**Table 2:** **Grading of protrusio acetabuli according to the distance between the acetabular line (medial wall of acetabulum) and the ilio-ischial line**^^**25**^^

**Grade**	**Men**	**Women**
I	3-8 mm	6-11 mm
II	8-13 mm	12-17 mm
III	>13 mm with fragmentation	>17 mm with fragmentation

## Total knee replacement

**Figure 3:**
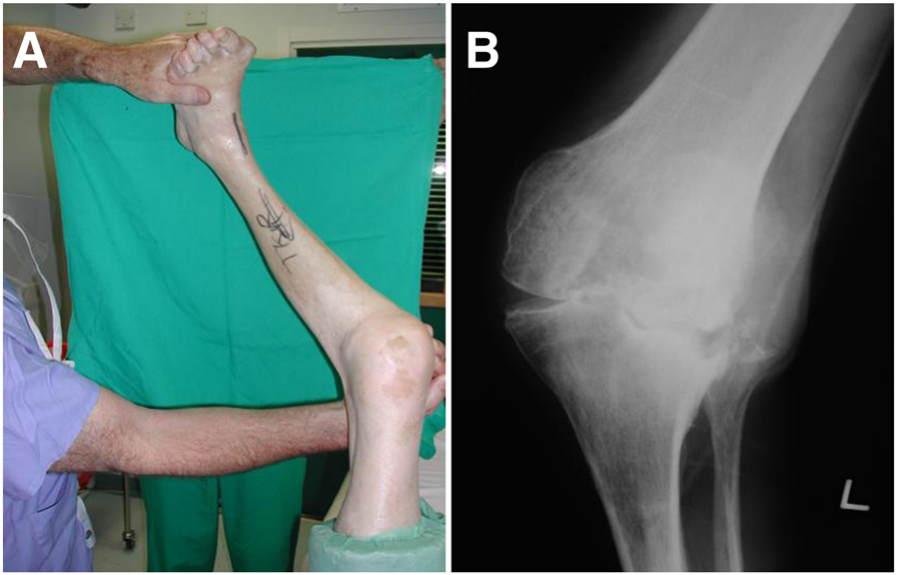
Valgus deformity of knee (A) due to avascular necrosis and bone destruction (B).

Poor bone stock, avascular necrosis, deformity and contracture (Figure 3) can present technical challenges. Implant augmentation and bone grafting may be required.

A posterior cruciate ligament (PCL) retaining implant is favoured by many surgeons for osteoarthritis of the knee. However, in rheumatoid disease there is soft tissue destruction resulting in joint instability. Even if the PCL is intact intra-operatively this may subsequently be eroded by inflammatory pannus post-operatively resulting in an unstable prosthetic joint. Laskin reviewed 178 rheumatoid patients at an average of 8.2 years follow-up and demonstrated a 50% instability rate with PCL retaining implants in contrast to a 1% instability rate with the PCL sacrificing implants [24]. Longer-term results in rheumatoid disease are limited, Goldberg et al. [25] and Kristensen et al. [26] demonstrated a 0% to 14% instability rate for PCL sacrificing implants respectively. Gill et al. [27] and Meding et al [28] have also shown similar rates of instability for PCL retaining implants (1.5% and 9.9% respectively). The differences between the reported instability rates may relate to disease severity and medical treatment, with more recent studies having the advantage of modern pharmacokinetics and preservation of soft tissues.

**Figure 4:**
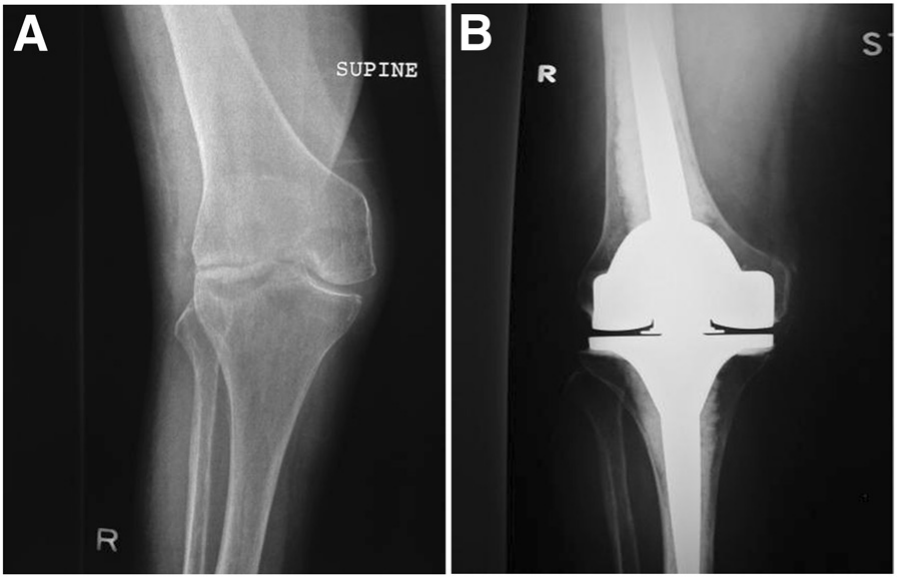
Significant valgus deformity and concomitant medial collateral attenuation (A) managed with a rotating hinge TKR (B).

Patients with significant valgus deformity and concomitant medial collateral attenuation, a rotating hinge (Figure 4) may be the treatment of choice [29], as an extensive lateral release may result in “overstuffing” of the joint with an increased risk of mid-flexion instability. Furthermore if the patient has a marked fixed flexion contracture of >30 degrees, then threshold for a constrained design should be low, particularly in the elderly patient [30].

The 15 year survival excluding infection for cemented total knee arthroplasty in RA is 96.5% and 91% for PCL retaining and PCL sacrificing implants respectively [28,31]. However, it could be argued that the increased failure rate in those who received a PCL sacrificing implant had a higher grade of rheumatoid disease with severe joint destruction and hence the indication of a stabilised implant. Cemented implants may be the preferred option in poor bone stock and osteoporosis. Vigano et al described a 10-year survival rate of 98.4% using an uncemented TKR for RA patients. The average age of their cohort was 49.5 years; and it could be argued that these patients had a better bone stock than elderly patients facilitating osteointegration.

Shoji et al conducted a retrospective comparison of rheumatoid patients undergoing TKR with and without patella resurfacing and demonstrated no difference in pain or functional outcome [32]. In contrast Kajino et al conducted a prospective randomised control trial of rheumatoid patients undergoing TKR and demonstrated improved pain relief and functional outcomes for patients receiving patella resurfacing [33].

## Total ankle replacement

The survival of total ankle replacement (TAR) does not parallel that of THR and TKR. The reported success rate of TAR in RA ranges from 40 to 100% [34]. Mechanical loosening of the components is the major cause of revision surgery [35]. A recent long-term follow-up of 33 TAR for RA reported an 85% survival rate at 10 years when failure was defined as removal of the prosthesis, this decreased to 64% if signs of radiographic loosening were included [35].

Failure after TAR has been shown to be much higher in patients with greater than fifteen degrees of varus or valgus deformity [36,37]. When a concomitant planovalgus, forefoot abductus deformity exists, arthroplasty is a more difficult and is a less predictable procedure. Success will require a simultaneous, or two-stage triple arthrodesis to correct the deformity, which is generally too severe to be corrected with a simple subtalar fusion. Patients are often frail or have poor soft tissues making two stage operations unattractive and a simultaneous triple arthrodesis a high risk venture.

## Revision arthroplasty surgery

There is limited data regarding revision THR in rheumatoid patients. The outcome of cemented cup revision for RA is inferior to patients without RA with a 64% radiographic failure rate at 7 years [38]. This survival rate falls further at 9 years to 44% when an uncemented cup is used at revision [39]. Schreurs et al improved survival with the application of morselised bone graft in combination with a cemented cup at revision, reporting an 80% survival at 12 years [40].

High failure rates have been reported for revision TKR in patients with RA. Garcia et al. report a survival for all knees (27 mechanical failures and 18 infected revisions) of 76% at 5 years. They also, more worryingly, report a 34 % mortality rate at 6 months for RA patients revised for infection [41].

## Rehabilitation

Patients with RA have a longer length of hospital stay with slower functional improvement than patients undergoing joint replacement surgery for primary osteoarthritis. A study of 1,361 rheumatoid patients and 26,096 osteoarthritic patients undergoing lower limb arthroplasty found the length of stay to be only one day longer, but did show a slower, more gradual improvement of their functional independence score [42]. Stanley et al demonstrated that RA patients undergoing bilateral TKR had a similar functional outcome and complication rate as those undergoing staged procedures, but they had the benefit of a more rapid recovery relative to staged procedures [43].

## Complications

Evidence from the Swedish joint registry suggests that periprosthetic fractures are more common among patients with rheumatoid disease compared to osteoarthritis patients, with a hazard ratio (HR) of 1.56 [44]. Similar figures have been reported from the Finnish registry (HR 2.1) [45]. This predisposition to fracture may to be secondary to poor bone quality [45]. The management of peri-prosthetic fractures can be challenging and associated with high morbidity and mortality [43].

The risk of arthroplasty infection is greater for patients with RA. Bongartz et al conducted a retrospective review of 462 patients (657 implants) who received either a TKR or THR, they compared infection rates for both RA patients and with a matched cohort of patients with osteoarthritis [46]. They found RA patients to be at an increased risk of prosthetic joint infections for both primary (HR 4.08, 95% CI 1.35-12.33) and revision surgery (HR 2.99, 95% 1.02-8.75).

Conflicting evidence exists regarding the risk of venous thromboembolism (VTE) post arthroplasty surgery in RA, with Chotanaphuti et al. [47] declaring RA to be a risk factor and Guan et al. [48] claiming RA to be protective for VTE. A retrospective review of nearly 5 million patients with RA showed that RA was an independent risk factor for pulmonary embolism and deep vein thrombosis in hospital patients who did not undergo surgery, with a relative risk of 2.25 and 1.9 respectively [49].

## Patient outcomes

Those patients with active disease, raised rheumatoid titre or clinical depression do not improve to the same extent as those patients without [50]. Ethgen et al performed a cost/outcome analysis of arthroplasty for patients with RA finding good pain relief that was equal to those with primary osteoarthritis, but there was only a minor improvement in the functional outcome [51]. They also demonstrated reduced use of DMARDS, with cost savings, which may relieve the patient of their side-effects. Sledge proposed the key to a successful surgical outcome for patients with RA is for the surgeon to be familiar with the technical challenges of patients with polyarthritis and to work as part of a multidisciplinary team [1].

## Summary

RA is a systemic disease and like any other medical comorbidity, the patient should be optimised pre-operatively using a multidisciplinary approach. The continued use of methotrexate does not increase infection risk, and aids an early recovery with control of the disease during the peri-operative period. Biologic agents (TNFα antagonists) should be stopped pre-operatively due the increased infection rate. Patients should be made aware pre-operatively of the increased risk of infection and periprosthetic fracture rates associated with their disease.

The surgical sequence is commonly hip, knee and then ankle. Cemented THR and TKR have superior survival rates over uncemented components in RA patients. The need for bone grafting for protrusio acetabuli should be identified during pre-operative planning. The evidence is not clear regarding a PCL sacrificing versus retaining in TKR, but a PCL sacrificing component limits the risk early instability and potential revision. Patella resurfacing as part of a TKR is associated with improved outcomes and should be considered in the rheumatoid patient. The results of TAR remain inferior to THR and TKR. RA patients achieve equivalent pain relief, but their rehabilitation is slower and their functional outcome is not as good. However, the key to managing these complicated patients is to work as part of a multidisciplinary team to optimise their outcome.

## Competing interests

The authors declare that they have no competing interests.

## Author’s contributions

NDC conducted a literature review, analysed the data, and composed the paper. SJB and LCB, as senior authors and experts in lower limb arthroplasty, were involved in editing the final manuscript and given approval to the final version submitted for publication. All authors have read and approved the final manuscript.

## References

[B1] Sledge CB Saunders WB Introduction to surgical management of patients with arthritis Kelly's Textbook of rheumatology 2001 Philadelphia

[B2] Macgregor AJ Snieder H Rigby AS Koskenvuo M Kaprio J Aho K Silman AJ Characterizing the quantitative genetic contribution to rheumatoid arthritis using data from twins Arthritis Rheum 2000 43 30 37 10.1002/1529-0131(200001)43:1<30::AID-ANR5>3.0.CO;2-B 10643697

[B3] Scott DL Symmons DP Coulton BL Popert AJ Long-term outcome of treating rheumatoid arthritis: results after 20 years Lancet 1987 1 1108 1111 2883443 10.1016/s0140-6736(87)91672-2

[B4] Louie GH Ward MM Changes in the rates of joint surgery among patients with Rheumatoid Arthritis in California, 1983–2007 Ann Rheum Dis 2010 69 5 868 871 10.1136/ard.2009.112474 19581279 PMC2859109

[B5] James D Young A Kulinskaya E Knight E Thompson W Ollier W Dixey J Orthopaedic intervention in early rheumatoid arthritis. Occurrence and predictive factors in an inception cohort of 1064 patients followed for 5 years Rheumatology (Oxford) 2004 43 369 376 14722346 10.1093/rheumatology/keh059

[B6] http://www.ncbi.nlm.nih.gov/sites/entrez

[B7] Wilkinson JM Stanley D Getty CJM Surgical management of the rheumatoid patient Curr Orthop 2004 18 357 370 10.1016/j.cuor.2004.06.007

[B8] Conlon PW Isdale IC Rose BS Rheumatoid arthritis of the cervical spine. An analysis of 333 cases Ann Rheum Dis 1966 25 120 126 5931006 10.1136/ard.25.2.120 PMC2453383

[B9] Roche CJ Eyes BE Whitehouse GH The rheumatoid cervical spine: signs of instability on plain cervical radiographs Clin Radiol 2002 57 241 249 10.1053/crad.2001.0745 12014867

[B10] Takatori R Tokunaga D Hase H Mikami Y Ikeda T Harada T Imai K Nishimura T An HS Inoue N Kudo T Three-dimensional morphology and kinematics of the craniovertebral junction in rheumatoid arthritis Spine 2010 35 1278 1284 10.1097/BRS.0b013e3181e6d578 20736886

[B11] Anstead GM Steroids, retinoids, and wound healing Adv Wound Care 1998 11 277 285 10326344

[B12] Weinblatt ME Maier AL Fraser PA Coblyn JS Longterm prospective study of methotrexate in rheumatoid arthritis: conclusion after 132 months of therapy J Rheumatol 1998 25 238 242 9489813

[B13] Grennan DM Gray J Loudon J Fear S Methotrexate and early postoperative complications in patients with rheumatoid arthritis undergoing elective orthopaedic surgery Ann Rheum Dis 2001 60 214 217 10.1136/ard.60.3.214 11171680 PMC1753573

[B14] Chen YF Jobanputra P Barton P Jowett S Bryan S Clark W Fry-Smith A Burls A A systematic review of the effectiveness of adalimumab, etanercept and infliximab for the treatment of rheumatoid arthritis in adults and an economic evaluation of their cost-effectiveness Health Technol Assess 2006 10 iii-xiii 1 10.3310/hta10420 17049139

[B15] Bibbo C Goldberg JW Infectious and healing complications after elective orthopaedic foot and ankle surgery during tumor necrosis factor-alpha inhibition therapy Foot Ankle Int 2004 25 331 335 15134615 10.1177/107110070402500510

[B16] Kawakami K Ikari K Kawamura K Tsukahara S Iwamoto T Yano K Sakuma Y Tokita A Momohara S Complications and features after joint surgery in rheumatoid arthritis patients treated with tumour necrosis factor-alpha blockers: perioperative interruption of tumour necrosis factor alpha blockers decreases complications? Rheumatology (Oxford) 2010 49 2 341 347 10.1093/rheumatology/kep376 19965973

[B17] Guidance on the use of metal on metal hip resurfacing arthroplasty 2002 London: National Institute for Clinical Excellence http://www.nice.org.uk/nicemedia/pdf/HipResurfacing-FinalGuidance.pdf

[B18] Chmell MJ Scott RD Thomas WH Sledge CB Total hip arthroplasty with cement for juvenile rheumatoid arthritis. Results at a minimum of ten years in patients less than thirty years old J Bone Joint Surg Am 1997 79 44 52 9010185 10.2106/00004623-199701000-00005

[B19] Creighton MG Callaghan JJ Olejniczak JP Johnston RC Total hip arthroplasty with cement in patients who have rheumatoid arthritis. A minimum ten-year follow-up study J Bone Joint Surg Am 1998 80 1439 1446 9801212 10.2106/00004623-199810000-00005

[B20] Eskelinen A Paavolainen P Helenius I Pulkkinen P Remes V Total hip arthroplasty for rheumatoid arthritis in younger patients: 2,557 replacements in the Finnish Arthroplasty Register followed for 0–24 years Acta Orthop 2006 77 853 865 10.1080/17453670610013132 17260192

[B21] Havelin LI Hallan G Dybvik E Cemented verses uncemented femoral stems in rheumatoid arthritis patients in th Norwegian arthroplasty register European Federation of National Associations Of Orthopaedics and Traumatology (8th Congress) 2007 Vol. Florence 91-B ed 89 90

[B22] Sotelo-Garza A Charnley J The results of Charnley arthroplasty of hip performed for protrusio acetabuli Clin Orthop Relat Res 1978 12 8 679527

[B23] Hirst P Esser M Murphy JC Hardinge K Bone grafting for protrusio acetabuli during total hip replacement. A review of the Wrightington method in 61 hips J Bone Joint Surg Br 1987 69 229 233 3546328 10.1302/0301-620X.69B2.3546328

[B24] Laskin RS O'Flynn HM The Insall Award. Total knee replacement with posterior cruciate ligament retention in rheumatoid arthritis. Problems and complications Clin Orthop Relat Res 1997 24 8 9418617

[B25] Goldberg VM Figgie MP Figgie HE Heiple KG Sobel M Use of a total condylar knee prosthesis for treatment of osteoarthritis and rheumatoid arthritis. Long-term results J Bone Joint Surg Am 1988 70 802 811 3392077

[B26] Kristensen O Nafei A Kjaersgaard-Andersen P Hvid I Jensen J Long-term results of total condylar knee arthroplasty in rheumatoid arthritis J Bone Joint Surg Br 1992 74 803 806 1447237 10.1302/0301-620X.74B6.1447237

[B27] Gill GS Joshi AB Long-term results of retention of the posterior cruciate ligament in total knee replacement in rheumatoid arthritis J Bone Joint Surg Br 2001 83 510 512 10.1302/0301-620X.83B4.11398 11380120

[B28] Meding JB Keating EM Ritter MA Faris PM Berend ME Long-term followup of posterior-cruciate-retaining TKR in patients with rheumatoid arthritis Clin Orthop Relat Res 2004 146 52 10.1097/01.blo.0000147134.52561.64 15534535

[B29] Morgan H Battista V Leopold SS Constraint in primary total knee arthroplasty J Am Acad Orthop Surg 2005 13 515 524 16330513 10.5435/00124635-200512000-00004

[B30] Sculco TP The role of constraint in total knee arthroplasty J Arthroplasty 2006 21 54 56 10.1016/j.arth.2006.02.166 16781430

[B31] Rodriguez JA Saddler S Edelman S Ranawat CS Long-term results of total knee arthroplasty in class 3 and 4 rheumatoid arthritis J Arthroplasty 1996 11 141 145 10.1016/S0883-5403(05)80007-5 8648306

[B32] Shoji H Yoshino S Kajino A Patellar replacement in bilateral total knee arthroplasty. A study of patients who had rheumatoid arthritis and no gross deformity of the patella J Bone Joint Surg Am 1989 71 853 856 2745482

[B33] Kajino A Yoshino S Kameyama S Kohda M Nagashima S Comparison of the results of bilateral total knee arthroplasty with and without patellar replacement for rheumatoid arthritis. A follow-up note J Bone Joint Surg Am 1997 79 570 574 10.1302/0301-620X.79B4.7238 9111403

[B34] Michael JM Golshani A Gargac S Goswami T Biomechanics of the ankle joint and clinical outcomes of total ankle replacement J Mech Behav Biomed Mater 2008 1 276 294 10.1016/j.jmbbm.2008.01.005 19627793

[B35] Jensen NC Linde F Long-term follow-up on 33 TPR ankle joint replacements in 26 patients with rheumatoid arthritis Foot Ankle Surg 2009 15 123 126 10.1016/j.fas.2008.08.009 19635418

[B36] Hobson SA Karantana A Dhar S Total ankle replacement in patients with significant pre-operative deformity of the hindfoot J Bone Joint Surg Br 2009 91 481 486 10.1302/0301-620X.91B4.20855 19336808

[B37] Wood PL Crawford LA Suneja R Kenyon A Total ankle replacement for rheumatoid ankle arthritis Foot Ankle Clin 2007 12 497 508 10.1016/j.fcl.2007.05.002 17765841

[B38] Raut VV Siney PD Wroblewski BM Cemented revision Charnley low-friction arthroplasty in patients with rheumatoid arthritis J Bone Joint Surg Br 1994 76 909 911 7983117

[B39] Mont MA Domb B Rajadhyaksha AD Padden DA Jones LC Hungerford DS The fate of revised uncemented acetabular components in patients with rheumatoid arthritis Clin Orthop Relat Res 2002 140 8 10.1097/00003086-200207000-00018 12072756

[B40] Schreurs BW Thien TM de Waal Malefijt MC Buma P Veth RP Slooff TJ Acetabular revision with impacted morselized cancellous bone graft and a cemented cup in patients with rheumatoid arthritis: three to fourteen-year follow-up J Bone Joint Surg Am 2003 85-A 647 652 12672840 10.2106/00004623-200304000-00010

[B41] Garcia RM Hardy BT Kraay MJ Goldberg VM Revision total knee arthroplasty for aseptic and septic causes in patients with rheumatoid arthritis Clin Orthop Relat Res 2010 468 82 89 10.1007/s11999-009-1061-x 19727993 PMC2795816

[B42] Nguyen-Oghalai TU Ottenbacher KJ Caban M Granger CV Grecula M Goodwin JS The impact of rheumatoid arthritis on rehabilitation outcomes after lower extremity arthroplasty J Clin Rheumatol 2007 13 247 250 10.1097/RHU.0b013e3181570ad4 17921790

[B43] Stanley D Stockley I Getty CJ Simultaneous or staged bilateral total knee replacements in rheumatoid arthritis. A prospective study J Bone Joint Surg Br 1990 72 772 774 2211753 10.1302/0301-620X.72B5.2211753

[B44] Lindahl H Malchau H Oden A Garellick G Risk factors for failure after treatment of a periprosthetic fracture of the femur J Bone Joint Surg Br 2006 88 26 30 10.1302/0301-620X.88B1.17029 16365115

[B45] Sarvilinna R Huhtala HS Puolakka TJ Nevalainen JK Pajamaki KJ Periprosthetic fractures in total hip arthroplasty: an epidemiologic study Int Orthop 2003 27 359 361 10.1007/s00264-003-0493-2 12898199 PMC3461876

[B46] Bongartz T Halligan CS Osmon DR Reinalda MS Bamlet WR Crowson CS Hanssen AD Matteson EL Incidence and risk factors of prosthetic joint infection after total hip or knee replacement in patients with rheumatoid arthritis Arthritis Rheum 2008 59 1713 1720 10.1002/art.24060 19035425 PMC3923416

[B47] Chotanaphuti T Ongnamthip P Songpatanasil T Veerapan P Deeprecha K Risk factors of deep vein thrombosis (DVT) after total knee arthroplasty (TKA) at Phramongkutklao Hospital J Med Assoc Thai 2007 90 485 491 17427525

[B48] Guan ZP Lu HS Chen YZ Song YN Qin XL Jiang J Clinical risk factors for deep vein thrombosis after total hip and knee arthroplasty Zhonghua Wai Ke Za Zhi 2005 43 1317 1320 16271243

[B49] Matta F Singala R Yaekoub AY Najjar R Stein PD Risk of venous thromboembolism with rheumatoid arthritis Thromb Haemost 2009 101 134 138 19132199

[B50] Benjamin A Helal B Surgical repair and reconstruction in rheumatoid disease 1980 London: The Macmillan Press

[B51] Ethgen O Kahler KH Kong SX Reginster JY Wolfe F The effect of health related quality of life on reported use of health care resources in patients with osteoarthritis and rheumatoid arthritis: a longitudinal analysis J Rheumatol 2002 29 1147 1155 12064827

